# Restoring a synthetic methylxanthine degradation operon to its native genes

**DOI:** 10.17912/micropub.biology.002105

**Published:** 2026-05-21

**Authors:** Sandy T. Nguyen, Romanus N. Ike, Kyle O. Kamanu, Dennis M. Mishler

**Affiliations:** 1 Microbe Hackers Stream, The Freshman Research Initiative, College of Natural Sciences, The University of Texas at Austin, Austin, TX, US; 2 Department of Molecular Biosciences, The University of Texas at Austin, Austin, TX, US

## Abstract

Caffeinated Coli are *Escherichia coli* strains engineered to degrade methylxanthines, providing an accessible bioassay for determining methylxanthine concentrations, with applications in food safety, bioremediation, medicine, and STEM education. The Caffeinated Coli decaffeination operon encodes four N-demethylation enzymes, including three N-demethylases, from *Pseudomonas putida* CBB5. A fifth *P. putida* protein essential for N _7_ -demethylation, NdmE, was substituted with the homolog Gst9, which may have unfavorable interactions with the *P. putida* enzymes, compromising N _7_ -demethylation in Caffeinated Coli. Here, we restored *ndmE* to the decaffeination operon and found most of the new N _7_ -demethylating strains can grow at slightly higher temperatures than their *gst9* -containing counterparts.

**Figure 1. Caffeinated Coli bioassay and restoration of ndmE gene:** (A) Endpoint OD600 measurements of Caffeinated Coli cultures with known methylxanthine concentrations (top left) can be used to create a standard curve (right) for determining methylxanthine concentrations of unknown samples (bottom left). (B) The pDCAF-ABC plasmid contains four genes (blue) from *Pseudomonas putida* strain CBB5 (Yu et al., 2009) and one gene (red) from *Janthinobacterium* sp. Marseille (Quandt et al., 2013). The *ndmA* , *ndmB* , and *ndmC* genes encode demethylases that target the N _1_ -, N _3_ -, and N _7_ -methyl groups of methylxanthines, respectively. All three demethylases require NdmD as a reductase *.* N _7_ -demethylation additionally requires Gst9. (C) Alignment of Gst9 and NdmE protein sequences. Non-conserved residues are red. Conservation scores (yellow histogram) represent how many of 10 physico-chemical properties are conserved at each position in the alignment (Livingstone & Barton, 1993); shorter, darker bars indicate lower conservation scores. (D) Maximum temperature where methylxanthine-dependent growth is observed for each Caffeinated Coli strain (pDCAF in red or pDECAF in blue). Each strain was grown in the presence of the highest-order methylxanthine it demethylates, as described in the methods. Each circle represents one biological replicate. Unshaded circles indicate that some growth was observed at the temperature, but not to saturation.

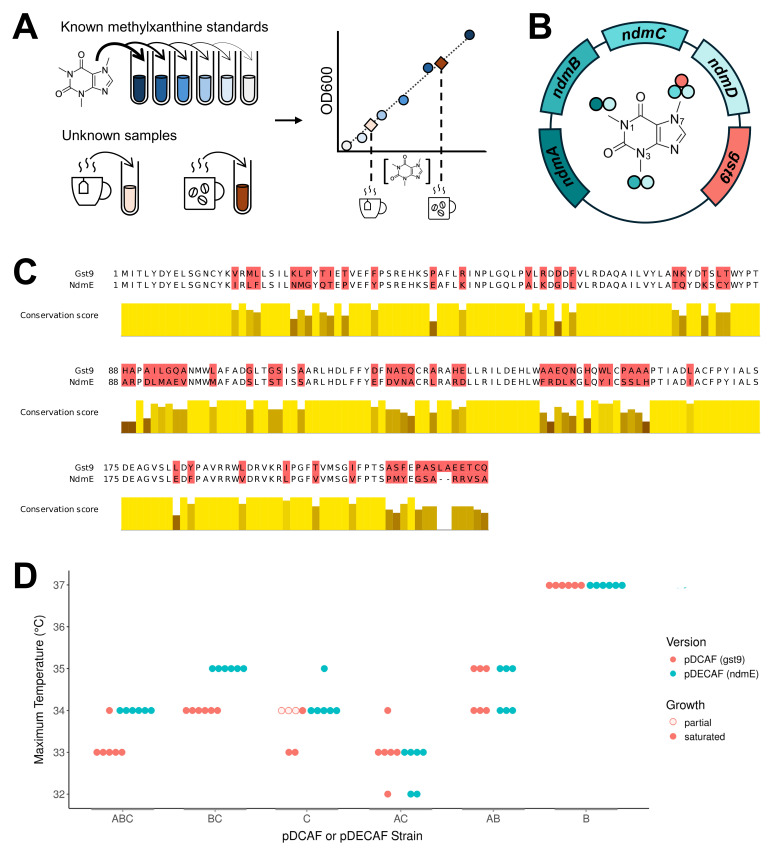



## Description

Methylxanthines, such as caffeine (1,3,7-trimethylxanthine) and theobromine (3,7-dimethylxanthine), are stimulant molecules that are found in foods and beverages, have therapeutic value, are markers of anthropogenic contamination in natural waters, and can have negative environmental impacts (Buerge et al., 2003; Oñatibia-Astibia et al., 2016; Mock & Summers, 2024). Quantifying methylxanthines is therefore relevant to a variety of areas, including food science, pharmaceutical manufacturing, and environmental remediation. However, standard analytical chemistry methods for quantification, such as HPLC, require laborious sample preparation and expensive equipment.

We previously developed a suite of *E. coli* strains, called Caffeinated Coli, as a more accessible method for methylxanthine quantification (Quandt et al., 2013; Gutierrez et al., 2019). These strains derive from the Keio collection of single gene knockouts (Baba et al., 2006): they lack the *guaB* gene, which is essential for de novo guanine biosynthesis. Additionally, they have been engineered to degrade specific methylxanthines into xanthine. The cells then use this xanthine in a salvage pathway to produce guanine, thus coupling growth to methylxanthine availability in minimal media. When methylxanthine is the limiting growth factor, methylxanthine concentrations are directly proportional to the maximum density (OD _600_ ) of a Caffeinated Coli culture, and a standard curve can be used to determine methylxanthine concentrations of unknown samples ( Figure 1A ). This bioassay's accuracy is comparable to HPLC (Gutierrez et al., 2019), and its portability, affordability, ease of use, and real-world relevance make it suitable for use in classrooms as an introduction to synthetic biology and genetic engineering (Tian et al., manuscript submitted).

The methylxanthine degradation ability of Caffeinated Coli originates from an N-demethylation (Ndm) operon found in *Pseudomonas putida* strain CBB5 (Yu et al., 2009). This *P. putida* operon includes *ndmA* , *ndmB* , and *ndmC* , which encode monooxygenases targeting the N _1_ -, N _3_ -, and N _7_ -methyl groups of methylxanthines, respectively ( Figure 1B ); *ndmD* , which encodes a reductase required for all three demethylation reactions; and *ndmE* , which encodes a putative glutathione-S-transferase required for N _7_ -demethylation (Quandt et al., 2013). Each Caffeinated Coli strain is defined by a pDCAF plasmid containing one, two, or three of the *ndmABC* genes; for example, the pDCAF-AB plasmid includes *ndmA* and *ndmB* , enabling the pDCAF-AB strain to fully demethylate and grow on 1,3-dimethylxanthine and 3-methylxanthine *.* However, because the 1-methyl position cannot be targeted by NdmA without the presence of an additional methyl group, the pDCAF-AB strain does not grow on 1-methylxanthine (Gutierrez, 2019). All pDCAF plasmids contain *ndmD* and, because the full coding sequence of *ndmE* was not available at the time, a homolog of *ndmE, gst9* , from *Janthinobacterium* sp. Marseille ( Figure 1B ; Quandt et al., 2013).

Caffeinated Coli are currently grown at 30°C because some of the N-demethylation reactions do not occur at 37°C in living cells. This is a non-optimal growth temperature for *E. coli* and increases the wait time for bioassay results. This temperature limitation of N-demethylation could be intrinsic to the N-demethylases, as they were sourced from bacteria found in ambient-temperature environments (Yu et al., 2009; Audic et al., 2007). However, the pDCAF-B strain, uniquely, grows at 37°C, demonstrating that at least N _3_ -demethylation can proceed at elevated temperatures. If demethylation of the N _3_ -methyl group can occur at 37°C, the other N-demethylation reactions may also be possible at this elevated temperature. We hypothesized that the thermal stability of N _7_ -demethylation, which uniquely requires a tightly bound NdmCDE complex, could be compromised by the replacement of NdmE with Gst9. In N _7_ -demethylation by *P. putida CBB5* , NdmE—functionally equivalent to Gst9 in Caffeinated Coli—is proposed to play a non-catalytic, structural role in forming the NdmCDE complex (Summers et al., 2013). NdmC, NdmD, and NdmE have high affinity for each other, maintained throughout the evolutionary history of *P. putida* CBB5, wherein the acquisition of a mutation in one of NdmC, NdmD, or NdmE would select for compensatory mutations in the others (Fraser et al., 2004). Since Gst9 has not coevolved with NdmCD, the interactions between Gst9 and NdmCD may be less favorable, thus reducing the stability of the complex responsible for N _7_ -demethylation in Caffeinated Coli relative to the native NdmCDE complex. When comparing the amino acid sequences, we find that Gst9 and NdmE have 67.4% sequence identity ( Figure 1C ), and while it is tempting to speculate that the differences between these proteins may be concentrated at their interfaces with NdmCD, the structure of the complex remains unsolved.

To test whether Gst9 compromises N _7_ -demethylation, we constructed a new suite of Caffeinated Coli strains in which *gst9* was replaced with *ndmE* . We hypothesized that this replacement might improve the thermal stability of N _7_ -demethylation, as a higher-affinity protein complex would require more thermal energy for dissociation. We found that for most of the N _7_ -demethylating Caffeinated Coli strains (ABC, BC, and C), the new *ndmE* -containing (pDECAF) versions were able to grow at slightly higher temperatures than their *gst9* (pDCAF) counterparts, consistent with our hypothesis. This difference was not observed for the non-N _7_ -demethylating strains (AB, B; Figure 1D ), supporting our interpretation that the observed differences are due to the presence of NdmE in place of Gst9 in the NdmCDE complex, which is responsible for N _7_ -demethylation activity. The pDECAF and pDCAF versions of each strain were grown in the same incubator at the same time, with randomized spatial arrangement of individual tubes, and the experiment was conducted twice per strain; thus, differences in maximum growth temperature between strain versions were not caused by temperature gradients or other fluctuations in incubator temperature.

Surprisingly, we observed no significant difference in thermal stability between the AC strains, suggesting that N _1_ -demethylation—which precedes N _7_ -demethylation (Mock et al., 2021)—is the limiting step in degradation of 1,7-dimethylxanthine at elevated temperatures. When comparing N _1_ -demethylating strains with their non-N _1_ -demethylating counterparts, we see additional evidence that N _1_ -demethylation is limiting: the pDCAF-AB and pDECAF-AB strains both have lower maximum growth temperatures than the pDCAF-B and pDECAF-B strains, respectively. This pattern is also present, to a lesser degree, when comparing both ABC and AC strains to their BC and C counterparts. These findings could be explained by the substrate affinities of NdmA and NdmB: at 30°C, in vitro, NdmA has higher affinity for 1,3,7-trimethylxanthine than 1,7-dimethylxanthine ( *K _m_* = 37 μM vs 53 μM), and NdmB has even higher affinity for its major substrates ( *K _m_* = 25 μM for 3,7-dimethylxanthine and 22 μM for 3-methylxanthine) (Summers et al., 2012). These differences may be exacerbated when higher-than-optimal temperatures disrupt enzyme-substrate binding.

Overall, our results indicate that restoring the N-demethylation operon with its native *ndmE* gene does improve the thermal stability of N _7_ -demethylation in vivo, although not as much as hoped. This work demonstrates the relevance of a protein's biochemical and evolutionary context to its function in heterologous settings; as such, design choices in synthetic biology should consider these factors. While the new pDECAF strains did not achieve growth at 37°C, they provide an improved starting point for additional efforts, such as rational engineering or directed evolution of the Ndm proteins, to make Caffeinated Coli functional at 37°C. Research, industrial, and teaching labs would find this commonly-used incubator temperature more convenient for conducting the bioassay, and faster growth at 37°C would enable more rapid quantification of methylxanthine-containing samples.

## Methods

***Strain construction.*** The *ndmE* coding sequence was retrieved from NCBI GenBank (KC778191). NdmE was aligned to Gst9 with Clustal Omega, and the alignment ( Figure 1C ) was visualized with Jalview (Waterhouse et al., 2009). To create a pDE entry vector as the backbone for all *ndmE* -containing (pDECAF) plasmids, a gBlock (Integrated DNA Technologies) containing the *ndmD* and *ndmE* genes downstream of a BsaI restriction site, with 5' and 3’ BsmBI sites, was inserted into the pYTK001 entry vector (Lee et al., 2015; AddGene: 65108) via BsmBI Golden Gate assembly. Subsets of the *ndmA* , *ndmB* , and *ndmC* genes were PCR-amplified from the appropriate pDCAF template plasmids (see Reagents), with primers adding 5’ and 3’ BsaI sites for BsaI Golden Gate assembly into pDE. Prior to PCR, a BsaI site in the *ndmA* gene was removed by introducing a silent mutation to the pDCAF template plasmids via Q5 site-directed mutagenesis (New England Biolabs).

All Golden Gate assembly reactions used 1x T4 DNA ligase buffer, 1 µL T4 ligase, 1 µL BsmBI (for assembly of pDE) or BsaI enzyme (for assembly of the pDECAF plasmids), and a 2:1 molar ratio of the insert DNA fragment (gBlock or purified PCR product) to the entry vector plasmid. Thermal cycling conditions were: 30x (42 °C (BsmBI) or 37°C (BsaI) 1.5 min, 16°C 3 min); 42° C or 37°C 10 min; 60°C 5 min. 5 µL of the Golden Gate assembly reactions were transformed into 50 µL chemically competent *E. coli* cells (DH5α for sequence verification or BW25113Δ *guaB* for Caffeinated Coli strains).

***Thermal stability assay.*** Liquid cultures for each strain used 3 mL M9CG medium (1x M9 minimal salts (BD Biosciences), 1x trace elements solution (134 µM EDTA, 31 µM FeCl _3_ , 6.2 µM ZnCl _2_ , 720 µM CuCl _2_ , 420 pM CoCl _2_ , 1.62 µM H _3_ BO _4_ , 81 pM MnCl _2_ ), 1 mM MgSO _4_ , 0.3 mM CaCl _2_ , 1 mg/L thiamin, 0.2% glucose, and 2 g/L casamino acids (BD Biosciences)), with 50 µg/mL kanamycin and 20 µg/mL chloramphenicol. Additionally, each strain was supplemented with 50 µM of its highest-order methylxanthine (MX): 1,3,7-MX for ABC strains, 3,7-MX for BC, 7-MX for C, 1,7-MX for AC, 1,3-MX for AB, and 3-MX for B. MX stocks were created as previously described (Gutierrez et al. 2019). Starter cultures were inoculated with individual colonies and incubated at 30°C (200-220 rpm) until saturation. Cultures were passaged 1:1000 into fresh media and incubated at increasing temperatures (1 °C increments), until saturation or up to 48 hours at each temperature. A strain’s maximum temperature ( Figure 1D ) was defined as the temperature above which no growth was observed. Data comprise two experiments of three biological replicates for each strain. The pDCAF and pDECAF versions of a strain were always grown at the same time, with tubes arranged randomly in the same incubator.

## Reagents

Plasmids

**Table:** 

Plasmid	Genotype	Source
pDECAF-ABC	* ColE1 ndmA ndmB ndmC ndmD ndmE Cam ^R^*	This study
pDECAF-BC	* ColE1 ndmB ndmC ndmD ndmE Cam ^R^*	This study
pDECAF-C	* ColE1 ndmC ndmD ndmE Cam ^R^*	This study
pDECAF-AC	* ColE1 ndmA ndmC ndmD ndmE Cam ^R^*	This study
pDECAF-AB	* ColE1 ndmA ndmB ndmD ndmE Cam ^R^*	This study
pDECAF-B	* ColE1 ndmB ndmD ndmE Cam ^R^*	This study
pDE	* ColE1 ndmD ndmE Cam ^R^*	This study
pDCAF-ABC	* pMB1 ndmA ndmB ndmC ndmD gst9 Cam ^R^*	AddGene 113652
pDCAF-BC	* pMB1 ndmB ndmC ndmD gst9 Cam ^R^*	AddGene 113651
pDCAF-C	* pMB1 ndmC ndmD gst9 Cam ^R^*	AddGene 113648
pDCAF-AC	* pMB1 ndmA ndmC ndmD gst9 Cam ^R^*	AddGene 113650
pDCAF-AB	* pMB1 ndmA ndmB ndmD gst9 Cam ^R^*	AddGene 113649
pDCAF-B	* pMB1 ndmB ndmD gst9 Cam ^R^*	AddGene 113647
